# A transcriptomic analysis of the adult stage of the bovine lungworm, *Dictyocaulus viviparus*

**DOI:** 10.1186/1471-2164-8-311

**Published:** 2007-09-05

**Authors:** Shoba Ranganathan, Shivashankar H Nagaraj, Min Hu, Christina Strube, Thomas Schnieder, Robin B Gasser

**Affiliations:** 1 Department of Chemistry and Biomolecular Sciences, Macquarie University, Sydney, New South Wales 2109, Australia; 2 Biotechnology Research Institute, Macquarie University, Sydney, New South Wales 2109, Australia; 3 Department of Veterinary Science, The University of Melbourne, 250 Princes Highway, Werribee, Victoria 3030, Australia; 4 Institute for Parasitology, University of Veterinary Medicine Hannover, Buenteweg 17, D-30559 Hannover, Germany

## Abstract

**Background:**

Lungworms of the genus *Dictyocaulus *(family Dictyocaulidae) are parasitic nematodes of major economic importance. They cause pathological effects and clinical disease in various ruminant hosts, particularly in young animals. *Dictyocaulus viviparus*, called the bovine lungworm, is a major pathogen of cattle, with severe infections being fatal. In this study, we provide first insights into the transcriptome of the adult stage of *D. viviparus *through the analysis of expressed sequence tags (ESTs).

**Results:**

Using our EST analysis pipeline, we estimate that the present dataset of 4436 ESTs is derived from 2258 genes based on cluster and comparative genomic analyses of the ESTs. Of the 2258 representative ESTs, 1159 (51.3%) had homologues in the free-living nematode *C. elegans*, 1174 (51.9%) in parasitic nematodes, 827 (36.6%) in organisms other than nematodes, and 863 (38%) had no significant match to any sequence in the current databases. Of the *C. elegans *homologues, 569 had observed 'non-wildtype' RNAi phenotypes, including embryonic lethality, maternal sterility, sterility in progeny, larval arrest and slow growth. We could functionally classify 776 (35%) sequences using the Gene Ontologies (GO) and established pathway associations to 696 (31%) sequences in Kyoto Encyclopedia of Genes and Genomes (KEGG). In addition, we predicted 85 secreted proteins which could represent potential candidates for developing novel anthelmintics or vaccines.

**Conclusion:**

The bioinformatic analyses of ESTs data for *D. viviparus *has elucidated sets of relatively conserved and potentially novel genes. The genes discovered in this study should assist research toward a better understanding of the basic molecular biology of *D. viviparus*, which could lead, in the longer term, to novel intervention strategies. The characterization of the *D. viviparus *transcriptome also provides a foundation for whole genome sequence analysis and future comparative transcriptomic analyses.

## Background

Parasitic nematodes of livestock cause substantial economic losses due to poor productivity, failure to thrive and deaths [1,2]. The financial losses associated with these endoparasites are estimated at billions of dollars per annum [3]. Lungworms of the genus *Dictyocaulus *(family Dictyocaulidae) are key parasitic nematodes which cause pathological effects and clinical disease in different ruminant hosts, particularly in young animals [4,5]. *Dictyocaulus viviparus*, the bovine lungworm, causes a severe and frequently fatal bronchitis (known colloquially as 'husk') which is of major importance in many countries [6]. Severe cases of dictyocaulosis lead to emphysema and pneumonia – heavy infections can cause a mortality rate of >20% among affected cattle [1,2].

*Dictyocaulus viviparus *has a direct life cycle [7]. The adult stages (females and males) live in the bronchi, where the ovoviviparous females produce eggs from which first-stage larvae (L1) usually hatch rapidly whilst in the lung or the intestinal tract. The L1s are then shed in the faeces of the bovine host. Under favourable environmental conditions, L1s develop through to the infective third-stage larvae (L3s) during a period of ~4–6 days. After ingestion by the host, L3s migrate through the gut wall to the mesenteric lymph nodes, moult, and, as fourth-stage larvae (L4s), are transported to the lungs. L4s penetrate the alveoli, moult and then develop into adults. However, larval stages can remain inhibited in the lungs for up to 5 months. In cattle, the period from ingestion of L3s to reproductive maturity of the adult worms is 3–4 weeks.

While there is considerable knowledge of the morphological changes taking place during the life cycle of *D. viviparus*, very little is understood about the fundamental molecular and biochemical processes underlying the development and survival of this parasite and the parasite-host interplay. Insights into such processes are fundamentally important and could provide a basis for the identification of molecular targets for the rational design of nematocidal compounds, vaccines or/and for diagnosis. To date, studies of *D. viviparus *have been limited to individual genes and proteins. For instance, before the present study (March 2007), 323 gene sequences, 221 protein sequences and 229 research articles relating to *D. viviparus *were available in public databases.

Current technological advances in genomics provide exciting opportunities for exploring basic molecular biological and biochemical aspects of *D. viviparus *and related nematodes. For instance, expressed sequence tag (EST) data sets facilitate the prediction and categorization of key molecules, particularly those linked to development (both in pre-parasitic and parasitic stages), sexual differentiation and maturation, based on comparisons with other organisms for which sequence and functional genomic data sets are available. Also, the complete genome sequence of the free-living nematode *Caenorhabditis elegans *and the wealth of information on gene expression and function for this nematode [8,9] provide a means of evaluating homologues and orthologues [10], since *D. viviparus *cannot be maintained or propagated effectively *in vitro *for the functional testing of genes and gene products. Also, the potential of gene silencing techniques [11,12] provides a prospect for the functional analysis of molecules in this and other parasitic nematodes.

In the present study, we provide a first insight into the transcriptome of the adult stage of *D. viviparus via *EST sequencing and apply a newly established computational platform [13] for the clustering and comparative analyses of the data set against data available for a range of organisms, with an emphasis on the best characterized nematode, *C. elegans*. The representative ESTs from this dataset have been annotated functionally at the gene and protein levels to aid in assigning, in the main, gene ontologies, protein families and biochemical pathways. Such annotation techniques have enabled us to pin-point genes that could be considered in the development of intervention strategies. The present data provide a foundation for future investigations in areas, such as the stage-, sex- and tissue-specific gene transcription or expression, whole genome sequencing and proteomics of *D. viviparus*.

## Results and discussion

### General EST analysis

**Figure 1:**
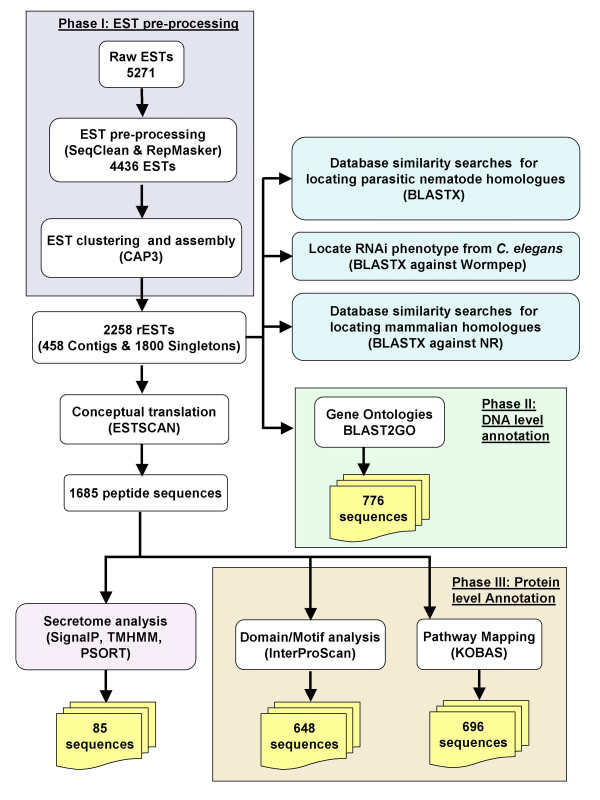
**Bioinformatics analysis of *D. viviparus *ESTs**. ESTExplorer analysis comprising Phases I (pre-processing), II (DNA-level annotation) and III (protein-level annotation), were augmented by homologue identification from nematodes as well as parasitic nematodes, using specialized databases.

Of 5271 clones sequenced, a total of 4436 quality ESTs were obtained (Phase I, Figure 1), achieving a sequencing success of 84% (Table 1), which is consistent with previous studies [14,15]. These pre-processed ESTs ranged from 80–1164 bp, with a mean of 730 bp and a standard deviation (S.D.) of 258 bp. After clustering, the mean length of the contigs (or consensus sequences) increased to 787 (+/- 313) bp. The G+C content of the coding sequences was 43.5%, consistent with other nematodes from clade V [16,17] and slightly more than C. *elegans *(37%) and its congener, *C. briggsae *(38%) [18]. Under the assumption that the *D. viviparus *genome codes for ~22,000 proteins [19], the EST clusters were predicted to represent ~10–15% of the proteins encoded by this genome.

**Table 1:** Preliminary analysis of the 5271 ESTs

	Numbers (percentage)
Raw sequences obtained	5271
Curated sequences	4436 (84)
Clusters of multiple sequences (contigs)	458 (8.6)
Clusters of singletons	1800 (34.2)
Total	2258 (42.8)

**Figure 2:**
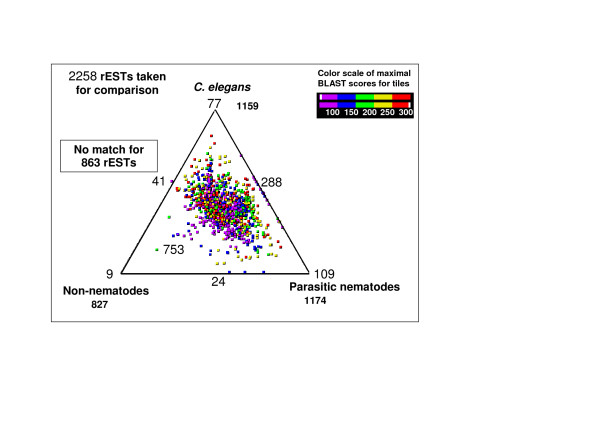
**Comparison of *D. viviparus *rESTs with *C. elegans*, parasitic nematode and non-nematode protein sequence databases using SimiTri**. The numbers at each vertex indicate the number of rESTs matching only that specific database. The numbers on the edges indicate the number of rESTs matching the two databases linked by that edge. The number within the triangle indicates the number of *D. viviparus *genes with matches to all three databases.

The cluster analysis of the 4436 ESTs from adult *D. viviparus *yielded 2258 representative ESTs (rESTs; 458 contig and 1800 singleton sequences; see Table 1), of which 1685 (74.6%) had open reading frames (ORFs). All rESTs were then subjected to analyses using ESTExplorer [13], a semi-automated bioinformatics pipeline (Fig. 1). Also, we queried rESTs (at the amino acid level) against three databases containing protein sequences from different organisms, in order to categorize the molecules from *D. viviparus*. Data were compared with protein sequences available for (i) *C. elegans *(from WORMPEP v.167 [20]), (ii) parasitic nematodes (available protein sequences and peptides from conceptually translated ESTs), and (iii) organisms other than nematodes (from NCBI non-redundant protein database) [21]. Three-way comparison of *D. viviparus *rESTs with homologues from *C. elegans*, WORMPEP and parasitic nematodes have been figuratively presented using SimiTri [22] (Figure 2). For this comparison, similarity searches of the 2258 representative sequences resulted in 1159 (51.3%) homologues to *C. elegans*, 1174 (51.9%) to those from other parasitic nematodes (including some strongylids), 827 (36.6%) homologues in organisms other than nematodes, and 39.5% had no significant similarity to any other organism (employing a cut-off of 1e-05) for which sequence data are currently available (Figure 2, Additional File 1). The SimiTri plot (Figure 2) shows that, given the current database contents, the sampled transcriptome from *D. viviparus *is equally close to available *C. elegans *and parasitic nematode sequences, compared with non-nematodes.

#### Comparative analyses with *C. elegans *data sets

The comparative analysis to identify homologues in *C. elegans *is important because *D. viviparus *and this free-living nematode are both considered to belong to clade V of the Nematoda [16,17], and because *C. elegans *also represents the best characterized nematode in many respects, particularly in terms of its genome, genetics, biology, physiology, biochemistry, as well as the localization and functions of molecules [20,23]. Specifically, the comparative analysis (at the amino acid level) of all rESTs with *C. elegans *data (see Additional File 1) revealed 1159 (51.3%) key, well-characterized molecules associated with various biological processes (n = 540), including development, regulation of biological processes, response to abiotic and biotic stimuli and reproduction. 'Non-wildtype' RNAi phenotypes in *C. elegans *(such as embryonic lethality, maternal sterility, sterility in progeny, larval arrest and slow growth) were associated with 569 (48.7%) of these 1159 molecules (Table 3). Of the 1159 *C. elegans *homologues, the functions for 776 (66%) rESTs could be inferred using Gene Ontologies (GO) [24], with 696 (41%) sequences being mapped to key biological pathways (including signal transduction mechanisms, antigen processing and presentation, regulation of actin cytoskeleton, ribosomal proteins and translation factors). Overall, the functional classification revealed that approximately half of the rESTs had homologues in *C. elegans *and parasitic nematodes, one tenth were specific to parasitic nematodes, and one third of the rESTs did not match any sequence in current databases, possibly representing novel genes.

**Table 3:** Gene Ontology mappings (using GO slim terms) for *Dictyocaulus viviparus *clusters. Note that individual GO categories can have multiple mappings

Categories and subcategories	Representation	% Representation of total
**a. Biological Process**	**540**	**8.53**
cellular process	277	4.38
cell communication	56	0.88
cell recognition	1	0.02
cell differentiation	33	0.52
response to stimulus	146	2.31
response to biotic stimulus	11	0.17
response to abiotic stimulus	30	0.47
response to stress	35	0.55
behavior	114	1.8
reproduction	180	2.84
growth	180	2.84
physiological process	424	6.7
response to endogenous stimulus	10	0.16
response to external stimulus	16	0.25
response to stress	35	0.55
death	35	0.55
metabolism	256	4.05
homeostasis	5	0.08
development	284	4.49
embryonic development	228	3.6
anatomical structure development	103	1.63
localization	54	0.85
**b. Cellular component**	**328**	**5.18**
cell	311	4.91
intracellular	264	4.18
organelle	215	3.4
vesicle	9	0.04
membrane-bound organelle	152	2.4
organelle envelope	1	0.02
non-membrane-bound organelle	86	1.36
organelle part	21	0.33
organelle lumen	18	0.28
envelope	1	0.02
protein complex	22	0.35
unlocalized protein complex	5	0.08
cell part	280	4.42
extracellular matrix (sensu Metazoa)	10	0.16
extracellular region	17	0.27
extracellular region part	15	0.24
extracellular space	5	0.08
**c. Molecular function**	**457**	**7.22**
binding	253	4
carbohydrate binding	4	0.06
ion binding	13	0.21
nucleotide binding	65	1.03
nucleic acid binding	80	1.26
chromatin binding	2	0.03
lipid binding	3	0.05
protein binding	107	1.69
antioxidant activity	2	0.03
catalytic activity	214	3.38
hydrolase activity	90	1.42
transferase activity	51	0.81
enzyme regulator activity	11	0.17
electron transporter activity	10	0.16
motor activity	1	0.02
signal transducer activity	12	0.19
receptor activity	4	0.06
structural molecule activity	214	1.69
transcription regulator activity	68	1.07
transporter activity	28	0.44
ion transporter activity	1	0.02
channel or pore class transporter activity	1	0.02
translation regulator activity	8	0.13

As multiple ESTs can be derived from the same gene, it was important to predict how many unique genes were represented by the rESTs. Mapping the 2258 *D. viviparus *rESTs to *C. elegans *revealed that, of the 1159 *D. viviparus *rESTs with similarities to 927 *C. elegans *genes, the majority of these (798/1159 or 68.8%) had a one-to-one relationship to their *C. elegans *homologue. The remaining 369 rESTs mapped to multiple non-overlapping regions from 129 *C. elegans *genes (with an estimated fragmentation rate of 25%). After discounting for fragmentation, we estimated that 1694 unique *D. viviparus *genes were identified, with a suggested new gene discovery rate of 38.2% (1694/2258).

*C. elegans *(non-wild-type) RNAi phenotypes can provide some indication of the relevance and functions of orthologous genes in other nematodes, particularly in parasitic nematodes of clade V, for which the complexity of an obligate parasitic life cycle and the lack of an effective (long-term) laboratory culture system make high-throughput functional screening impractical [25]. We retrieved *C. elegans *RNAi data representing *D. viviparus *homologues. Of 1159 *D. viviparus *rESTs, 569 had homologues in *C. elegans *which could be silenced by RNAi (Additional File 1). The RNAi phenotypes (as described by Wormbase) included Adl (adult lethal), Age (ageing alteration), Bmd (body morphology defect), Dpy (dumpy), Egl (egg laying defect), Emb (embryonic lethal), Gro (slow growth), Let (larval lethal), Lvl (larval lethal), Lva (larval arrest) and Unc (uncoordinated), whereas 590 homologues had no observable RNAi phenotype in *C. elegans*. We also found that 23% of the most abundant (Table 2) and 22% of the transcripts predicted to represent secreted proteins of *D. viviparus *(Additional File 2) had *C. elegans *homologues with non-wildtype RNAi phenotypes.

**Table 2:** The most abundant transcripts in adult *Dictyocaulus viviparus*

No	Cluster ID	Percentage	No of ESTs	Gene ID	E-value	% identity	Description from NR hit	*C. elegans *Homologue	Top hit in *C. elegans*	*C. elegans *RNAi	*C. elegans *gene ontology
1	DvContig 413	100	261	-	-	-	No significant similarity (novel)	No significant similarity (novel)	None	None	None
2	DvContig 438	94.64	247	CAF29502.1	6E-64	118/126 (93%)	major sperm protein *[Oesophagostomum dentatum]*	msp-76 – (Major Sperm Protein)	**ZK1251.6**	fat content increased	embryonic development; structural molecule activity
3	DvContig 329	31.03	81	ABA53863.1	2E-136	220/333 (66%)	cathepsin B-like cysteine protease 1 *[Parelaphostrongylus tenuis]*	cysteine protease	**F57F5.1**	embryonic lethal (Let) locomotion abnormal (unc) larval arrest (Lva)	cathepsin B activity
4	DvContig 371	27.20	71	**-**	-	-	No significant similarity (novel)	No significant similarity (novel)	None	None	None
5	DvContig 366	25.67	67	CAF29502.1	4E-65	118/126 (93%)	major sperm protein *[Oesophagostomum dentatum]*	msp-76 – (Major Sperm Protein)	**ZK1251.6**	fat content increased	embryonic development; structural molecule activity
6	DvContig 318	18.39	48	**-**	-	-	No significant similarity (novel)	No significant similarity (novel)	None	None	None
7	DvContig 238	16.48	43	AAO63577.1	3E-15	59/199 (29%)	secreted protein 5 precursor *[Ancylostoma caninum]*	vap-1 – (Venom-Allergen-like Protein)	**F11C7.3b**	No observed phenotype is found.	extracellular region
8	DvContig 419	13.03	34	**-**	-	-	No significant similarity (novel)	No significant similarity (novel)	None	None	None
9	DvContig 415	12.26	32	NP_500698.1	3E-26	142/237 (59%)	Sperm-Specific family, class Q family member (ssq-2) [*Caenorhabditis elegans]*	ssq-2 – (Sperm-Specific family, class Q)	**T28H11.5**	locomotion abnormal (unc)	locomotory behavior; GPCR protein signaling pathway; integral to membrane; leukotriene receptor activity; structural molecule activity
10	DvContig 140	11.88	31	NP_579952.2	7E-29	69/86 (80%)	cytochrome oxidase subunit I *[Ancylostoma duodenale]*	cytochrome oxidase subunit I	**MTCE.26**	No observed phenotype is found.	electron transport; membrane; cytochrome C-oxidase activity
11	DvContig 385	11.88	31	NP_500520.1	3E-62	230/300 (76%)	COLlagen family member (col-3) [*Caenorhabditis elegans*]	col-34 cuticular collagen	**F36A4.10**	organism morphology abnormal (Bmd), dumpy (Dpy), locomotion abnormal (unc)	locomotory behavior; GPCR protein signaling pathway; integral to membrane; leukotriene receptor activity; structural molecule activity
12	DvContig 357	9.96	26	NP_500697.1	2E-26	70/110 (63%)	Sperm-Specific family, class P family member (ssp-19) [*Caenorhabditis. elegans]*	ssp-11 – (Sperm-Specific family, class P)	**T28H11.6**	No observed phenotype is found.	structural molecule activity
13	DvContig 347	9.20	24	AAO63577.1	2.00E-15	59/199 (29%)	secreted protein 5 precursor *[Ancylostoma caninum]*	vap-1 – (Venom-Allergen-like Protein)	**F11C7.3b**	No observed phenotype is found.	extracellular region
14	DvContig 253	8.81	23	**-**	-	-	No significant similarity (novel)	No significant similarity (novel)	None	None	None
15	DvContig 261	7.66	20	AAO63577.1	6E-16	67/198 (33%)	secreted protein 5 precursor [*Ancylostoma caninum*]	testes-specific protein like	**T05A10.5**	No observed phenotype is found.	extracellular region
16	DvContig 352	7.66	20	NP_506519.1	1E-27	82/268 (30%)	Hypothetical protein CBG09313 *[Caenorhabditis briggsae] *Domain DUF856	T16A9.5 gene (Unnamed protein)	**T16A9.5**	No observed phenotype is found.	dicarboxylic acid transport
17	DvContig 390	7.66	20	**-**	-	-	No significant similarity (novel)	No significant similarity (novel)	None	None	None
18	DvContig 258	7.28	19	**-**	-	-	No significant similarity (novel)	No significant similarity (novel)	None	None	None
19	DvContig 403	7.28	19	**-**	-	-	No significant similarity (novel)	No significant similarity (novel)	None	None	None
20	DvContig 178	6.90	18	**-**	-	-	No significant similarity (novel)	No significant similarity (novel)	None	None	None
21	DvContig 86	5.75	15	AAO63577.1	1E-16	64/215 (29%)	secreted protein 5 precursor *[Ancylostoma caninum]*	testes-specific protein like	**T05A10.5**	No observed phenotype is found.	extracellular region
22	DvContig 346	5.75	15	**-**	-	-	No significant similarity (novel)	No significant similarity (novel)	None	None	None
23	DvContig 348	5.36	14	AAP41952.1	3E-12	55/203 (27%)	secreted protein ASP-2 *[Necator americanus]*	vap-1 – (Venom-Allergen-like Protein)	**F11C7.3b**	No observed phenotype is found.	None
24	DvContig 224	4.98	13	BAA12092.1	5E-168	310/366 (84%)	aldolase Ce2 [*Caenorhabditis elegans*]	Fructose-biphosphate aldolase	**F01F1.12a**	embryonic lethal (Let), sterile progeny (Stp), egg laying abnormal (Egl), sick (Sck), locomotion abnormal (unc), (Emb) slow growth (Gro)	None
25	DvContig 354	4.98	13	NP_510410.1	1E-16	49/77 (63%)	HIStone family member (his-24) [*Caenorhabditis elegans*]	his-24 histone H1	**M163.3**	No observed phenotype is found.	nucleosome assembly; DNA binding; nucleus
26	DvContig 216	4.60	12	CAE67138.1	7E-141	233/309 (75%)	casein kinase *[Caenorhabditis briggsae]*	casein kinase	**C39H7.1**	No observed phenotype is found.	protein amino acid phosphorylation; ATP binding; protein kinase activity; protein serine/threonine kinase activity; protein-tyrosine kinase activity
27	DvContig 161	4.21	11	AAO63577.1	3E-15	64/215 (29%)	secreted protein 5 precursor *[Ancylostoma caninum]*	vap-1 – (Venom-Allergen-like Protein)	**F11C7.3b**	No observed phenotype is found.	extracellular region
28	DvContig 202	4.21	11	**-**	-	-	No significant similarity (novel)	No significant similarity (novel)	None	None	None
29	DvContig 266	4.21	11	**-**	-	-	No significant similarity (novel)	No significant similarity (novel)	None	None	None
30	DvContig 356	4.21	11	NP_492448.1	3E-31	76/191 (39%)	C-type Lectin [*Caenorhabditis elegans*]	clec-87 – (C-type LECtin)	**C25A1.8**	No observed phenotype is found.	sugar binding activity
31	DvContig 154	3.83	10	NP_492457.1	0	785/852 (92%)	Elongation Factor family member (eft-2) [*Caenorhabditis elegans*]	eft-2 – (Elongation FacTor)	**F25H5.4**	embryonic lethal (Let), larval arrest (Lva, maternal sterile (Ste), protruding vulva (Pvl)	embryonic development; translational elongation; translational termination

#### Comparative analysis with data for other nematodes within clade V

*D. viviparus *is a member of clade V of the phylum Nematoda [16,17], which comprises members of the orders Strongylida, Rhabditida and Diplogasterida. We conducted a comparative analysis of all 2258 rESTs with data [17,26] for various nematodes (including *Ancylostoma caninum, Ancylostoma ceylanicum, Necator americanus, Nippostrongylus brasiliensis, Haemonchus contortus, Ostertagia ostertagi, Teladorsagia circumcincta *and *Pristionchus pacificus*) belonging to clade V (Additional File 3), to examine gene conservation within this clade. The analysis revealed 601 (26.6%) rESTs to have significant similarity to molecules from the members of clade V. These rESTs represented house-keeping (including cathepsin B-like cysteine proteases 1 and 2 and serine-threonine protein kinase) as well as nematode-specific (vitellogenin structural and major sperm protein) genes. We could assign GO terms to 461 of these 601 rEST sequences, associated with 118 different biological processes, such as embryonic development, intracellular protein transport, protein metabolism and responses to abiotic and/or biotic stimuli. Furthermore, 404 sequences could be mapped to biological pathways predicted to be associated with ribosomal proteins and the proteasome system, including 23 predicted secreted proteins mapping to cysteine proteinases, secreted protein 5 precursor and parasite pepsinogen.

#### Abundant transcripts in adult *D. viviparus*

A high level of representation in a cDNA library usually correlates with high transcript abundance in the original biological sample [27], although artefacts of library construction can result in a selection for or against representation of some transcripts. The *D. viviparus *clusters were ranked according to the number of contributing ESTs, and the top 30 clusters, which represented 1261 (24%) of the total number of rESTs (2258) obtained, were investigated in detail (Table 2). A number of clusters had significant alignments to known proteins, the majority of which were house-keeping genes, such as elongation factors, ribosomal proteins, aldolases, kinases, proteases and actins. Some of these genes have been identified in a number of other nematodes, including *Ancylostoma caninum*, *Ancylostoma ceylanicum, Dirofilaria immitis*, *Strongyloides ratti *and *Meloidogyne incognita *[28-32], requiring detailed characterisation to understand their functions in *D. viviparus*.

We analysed the most abundantly expressed transcripts from *D. viviparus *and found that 12 of the 30 (40%) contigs had no significant similarity to any sequence in the non-redundant protein database. As most of the nematode data are available only as ESTs and not included in the BLAST databases, we further compared these 12 contigs with sequences against Parasite Genomes using WU-Blast2 and BLASTN against the NCBI 'other-ESTs' database. We found that five sequences did not match any sequence in the databases, whereas seven entries had similarity to ESTs from other nematodes. The other 18 (60%) contigs were assigned functionality based on BLASTP against the NR database, and all of them had homologues in either non-parasitic (*C. elegans *and/or *C. briggsae*) or parasitic nematodes. A summary of these findings is provided in Table 2. Most of the homologues were found to be house-keeping or structural genes (including aldolase, histone family members, lectin, collagen and cytochrome oxidase), and four contigs were represented by molecules specific to nematodes, such as the major sperm proteins.

### Comparison with cDNAs from third-stage larvae of *D. viviparus*

Recently, Strube *et al. *[33] identified and characterized 28 cDNAs differentially transcribed between experimentally induced hypobiotic and infective third-stage larvae (L3) of *D. viviparus *using a suppressive-subtractive hybridization (SSH) approach. We compared these 28 sequences against our dataset of 2258 rESTs, using BLASTN, to identify whether any of them were represented in the adult stage, with only one match. The sole sequence common to both datasets was L3ni 18 (accession number EG374523), which matched EST D.viviparus_42_A11 in the present dataset, being a homologue of the *C. elegans *hypothetical protein C05D11.10 (mitochondrial/chloroplast ribosomal S17-like protein [code KOG3447]). Thus, this EST study of adult *D. viviparus *represents a novel dataset for parasitic nematodes in clade V, representing the Metastrongyloidea (cf. [47]).

### Functional classification of rESTs from adult *D. viviparus*

We annotated all 2258 rESTs systematically using a range of bioinformatic tools (details available in Figure 1). This annotation included functional classifications of rESTs using Gene Ontologies (GO) [24], pathway mapping using KEGG (Kyoto Encyclopedia of Genes and Genomes) [34], visualisation of EST data comparisons using SimiTri [22] and analyses of the *D. viviparus *secretome using SignalP [35], TMHMM [36] and PSORTII [37]. Results of these analyses are described in the following two sections

#### a. Gene Ontologies

Gene Ontology (GO) has been used widely to predict gene function and classification. GO provides a dynamic vocabulary and hierarchy that unifies descriptions of biological, cellular and molecular functions across genomes. We used BLAST2GO [38], a sequence-based tool to assign GO terms, extracting them for each BLAST hit obtained by mapping to extant annotation associations. We found that 776 (31%) of 2258 rESTs could be functionally assigned to biological processes (n = 540), cellular components (n = 328) and molecular functions (n = 457). A summary GO representation (using GO Slim) of the *D. viviparus *rESTs is given in Table 3.

Amongst the most common GO categories representing biological processes were: binding (GO: 0005488), catalytic activity (GO: 0003824) and structural molecule activity (GO: 0005198); development (GO: 0007275), metabolism (GO: 0008152), reproduction (GO:0000003) and growth (GO:0040007). The largest number of GO terms in cellular components was for cell part (GO:0044464), membrane-bound organelle (GO:0043227) and non-membrane-bound organelle (GO:0043228). A complete listing of GO mappings assigned for rESTs is provided in Additional File 4.

#### b. Pathway analysis using KEGG assignments

Biochemical functionality was predicted by mapping all 2258 rESTs to pathways, using KOBAS implemented within ESTExplorer [13], with an E-value cut-off of 1.0e-5. Enzyme commission (EC) numbers were used to appraise which sequences pertained to a specific pathway. A total of 696 (31%) sequences were mapped to 139 KEGG pathways, with 453 sequences representing metabolic enzymes characterized by unique EC numbers. The top 30 (highly represented) pathways are shown in Table 4.

**Table 4:** Top 30 selected metabolic pathways in adult *D. viviparus *mapped by Kyoto Encyclopedia of Genes and Genomes

Number	KEGG Pathway	rESTs sequence count	Enzymes
1	Signal transduction mechanisms	26	26
2	Glycolysis/Gluconeogenesis	24	24
3	Pyruvate metabolism	19	19
4	Regulation of actin cytoskeleton	19	1
5	Ribosome	19	0
6	Protein folding and associated processing	18	14
7	Focal adhesion	17	1
8	GTP-binding proteins	15	0
9	Insulin signaling pathway	14	11
10	Carbon fixation	12	12
11	Purine metabolism	12	12
12	Antigen processing and presentation	11	6
13	Glycerophospholipid metabolism	11	11
14	MAPK signaling pathway	11	3
15	Translation factors	10	4
16	Propanoate metabolism	9	9
17	Fructose and mannose metabolism	9	9
18	Citrate cycle (TCA cycle)	9	9
19	Butanoate metabolism	9	9
20	Tight junction	9	3
21	Wnt signaling pathway	9	3
22	Oxidative phosphorylation	9	9
23	Leukocyte transendothelial migration	8	0
24	Adherens junction	8	2
25	Ion channels	8	0
26	CD molecules	7	5
27	Adipocytokine signaling pathway	7	5
28	Proteasome	7	2
29	Valine, leucine and isoleucine degradation	7	7
30	Other enzymes	37	37

Molecules involved in signal transduction mechanisms (n= 26) and glycolysis/gluconeogenesis (h= 24) had the highest representation amongst the sequences mapped to KEGG pathways. We also identified 50 predicted proteins with potential roles in host-parasite interactions, with 11 molecules predicted to be involved in antigen processing and/or presentation, six in T-cell receptor signalling pathway and seven CD molecules. Although, at this stage, the precise role of such molecules in the parasite-host interplay is unclear, they could be involved in manipulating or evading the host's immune response(s) or associated with the parasite's innate immune response. Extensive experimental work would be required to test these proposals. Furthermore, we identified families of proteins representing serine, cysteine and metallo-proteinases as well as proteinase inhibitors (e.g., cystatins). While these enzymes are inferred to mediate or modulate proteolytic functions, which, in turn, may facilitate tissue migration and other interactions with host cells, the proteinase inhibitors may protect the parasite against digestion by host or endogenous proteinases excreted/secreted in the lung of the mammalian host [39]. A complete listing of the KEGG mappings is available as supplementary data (Additional File 5).

### Secretome analysis

An important starting point in the identification of potential novel drug or vaccine candidates in parasites is the prediction of molecules that are secreted or excreted in or around the host- parasite interface [40-42]. Examples of such proteins are the aspartyl protease inhibitor (API-1) [43], mi-msp-1 (similar to a venom allergen antigen AG5-like) [44] and the Ancylostoma-secreted protein (ASP) [45]. In the present data set (= 2258 rESTs), we identified 85 putatively secreted proteins representing a non-redundant catalogue of *D. viviparus *molecules (Additional 2). Of these, 26 (30.5%) sequences had no significant similarity to any sequence available in current databases, whereas 59 (69.4%) had homologues in nematodes, with 43 (50.5%) *C. elegans *and/or *C. briggsae *matches, and 16 (18.8%) homologues in various other parasitic nematodes, including the blood-feeding nematodes *Ancylostoma ceylanicum*, *Necator americanus *and *Haemonchus contortus*.

The secretome analysis (Additional File 2) revealed a number of unique features. Firstly, seven of the putative secreted protein entries were homologous to either sperm-specific family members [41] or major sperm proteins (MSP), consistent with the mass spectrometric analysis of secreted molecules from *D. viviparus *[41]. Secondly, acetylcholinesterase (AChE) has been identified as an important enzyme secreted by adult *D. viviparus*, thought to be involved in parasite survival in the host and, therefore, being a vaccine candidate [46]. We now report an entry for secreted acetylcholinesterase (D.viviparus_8_A01 of 332 amino acids, with a signal peptide length of 21) in the secretome analysis of the current EST dataset (Additional File 2), which may also represent a target for further characterization.

## Conclusion

The present study has given us a first glimpse of the transcriptome of the adult stage of the bovine lungworm, *D. viviparus*, and represents a starting point for studies in a number of different fundamental and applied areas. We used a comprehensive EST analysis pipeline, ESTExplorer, for this purpose, for functional annotation at the DNA and protein levels [13]. From this single study of 5271 ESTs, we have identified 55 novel sequences (1.4%), with high confidence, with no known homologue in any other nematode or mammal for which sequence data are presently available in public databases. These molecules are particularly interesting, as they may represent genes that may be specific to parasitism or to the species. However, such molecules are very challenging to work on, as their potential functions cannot be predicted using current bioinformatic approaches. However, there is considerable scope in exploring such molecules in the future, using a combination of genomic and proteomic approaches. Insights into such molecules and/or their interaction with the bovine host could provide opportunities for developing novel intervention approaches.

From a systematic viewpoint, *D. viviparus*, belongs to the Metastrongyloidea ("metastrongyles" or "lungworms") based on nuclear ribosomal DNA sequence data [47], as distinct from the Trichostrongyloidea (mainly in the stomach and small intestine), Strongyloidea (mostly in the large intestine) and Ancylostomatoidea (small intestine), and thus represents, from biological, host-parasite relationship and molecular evolutionary perspectives, a very interesting species for comparative genomic analysis with nematodes from these superfamilies. Therefore, this nematode brings a number of important benefits for future investigations, particularly for genome sequencing and for subsequent comparative evolutionary analyses. Indeed, *D. viviparus*, among other strongylid nematodes, has recently been selected for whole genome sequencing, to be carried out at the Genome Sequencing Center of Washington University in St Louis, USA [48]. For *D. viviparus*, the genomic information from the present study underpins future microarray analyses, focused on exploring the transcriptional profiles among different stages (e.g., larval *versus *adult stages; hypobiotic stages *versus *those which are not arrested; free-living (L1s and L2s) *versus *infective *versus *late larval stages), sexes (female *versus *male) and tissues (e.g., germline *versus *neural *versus *musculature *versus *intestine) of the parasite. Such studies, particularly those of the molecules differentially transcribed and expressed during the transition to parasitism, the invasion of the host and hypobiosis, could provide unique insights into such key molecular developmental and reproductive processes. While there is some controversy regarding the applicability and usefulness of RNAi to some parasitic nematodes, such as the Strongylida [11,12], comparative studies of gene-silencing and transgenesis in *C. elegans *are considered useful for exploring the function and regulation of some relatively conserved parasite genes, provided data are interpreted with caution [10]. This is particularly the case with the continued increase in genome sequence information.

With the future availability of whole genome sequence data for *D. viviparus*, it will also be possible to carry out meaningful mass spectroscopic analyses of differentially expressed proteins [49,50], allowing large-scale analysis of proteins from small amounts of parasite material. Such analyses will enable the link to be made between the regulation of transcription and translation and, importantly, in the study of parasites, will allow the analysis of proteins expressed within short time frames within or external to the host animal, or within organs or micro-environments within the parasite [51,52]. Hence, the application of an integrated bioinformatic-genomic-phenomic-proteomic ("systems biology") approach, focusing on developmental processes and mechanisms, could enhance our understanding of the molecular biology of moulting, invasion of and establishment in the host, hypobiosis (arrested development), and sexual differentiation, maturation and behaviour of *D. viviparus*. Clearly, progress in such fundamental areas could lead to the development of exciting new ways of treating, controlling or preventing this lungworm and other parasitic nematodes, by blocking or disrupting key biological pathways in them.

## Methods

### Parasite material

Adults of *D. viviparus *(strain HannoverDv2000) were produced in helminth-free male Holstein-Friesian calves (five months of age). Four weeks after oral inoculation with 3300 L3, the calves with patent *D. viviparus *infection were euthanized and the worms collected from the lungs as described by Wood *et al. *[53]. The worms were washed extensively (five times) in large volumes (100 ml) of fresh diethyl-pyrocarbonate (DEPC)-treated saline (22°C), transferred to sterile, RNase-free screw-top cryovials^® ^(4 ml; Roth, Karlsruhe, Germany) and frozen as 200 μl pellets in a minimal amount of saline at -75°C or -196°C until RNA isolation.

### Isolation of total RNA, cDNA synthesis and cDNA library construction

Total RNA was extracted from the adults of *D. viviparus *(under liquid nitrogen, employing a sterile mortar and pestle) using the TriPure isolation reagent^® ^(Roche Molecular Biochemicals). Integrity and yields of RNA were verified and estimated, respectively, using the Bioanalyzer 2100 (Agilent). Each RNA sample (~10 μg) was treated with 2 U of *DNase *I (Promega), incubated at 37°C for 30 min prior to heat denaturation of the enzyme (75°C for 5 min) and then stored at -70°C until use. The cDNA was produced from 10 μg of total RNA from *D. viviparus*, using an oligo (dT) primer and Superscript II reverse transcriptase (Invitrogen) and then purified over DyeEx columns (Qiagen).

A non-directional cDNA library was constructed in the plasmid vector pGEM-T (Promega) by T-ended cloning, according to the manufacturer's protocol. Colonies were screened using blue-white selection. Clones (n = 5271) were picked randomly and patched on to grided Luria Bertani (LB) agarose plates containing 100 mg/ml ampicillin. Single-pass sequencing (using the T7 primer) was performed employing BigDye Chemistry (v3.1) in a 3730 × l DNA analyser (Applied Biosystems).

### EST analysis

The ESTs were initially analysed and annotated using ESTExplorer, an automated EST analysis platform [13,54]. In brief, the analyses comprised three phases (see Figure 1). In phase I, all ESTs were pre-processed (SeqClean, RepeatMasker), aligned/clustered using the Contig Assembly Program CAP3, employing a minimum sequence overlap length "cut-off" of 30 bases and an identity threshold of 95% (in Phase I) for the removal of flanking vector and adapter sequences, followed by assembly. Phase II of the ESTExplorer led to gene ontology inference, at the DNA-level annotation, using BLAST2GO (V 1.6.2) [38], using Gene Ontology (MySQL-DB-data release go_200609). In Phase III, rESTs were then conceptually translated into peptides (using ESTScan), which were further characterized *via *InterProScan (domain/motifs), and peptides mapped to respective pathways in *C. elegans *using KOBAS (KEGG Orthology-Based Annotation System, KEGG data release 40.0). We also retrieved KO data from KEGG and then classified enzymes from non-enzymes based on our pathway mapping results. Peptides predicted from rEST were also compared, using BLASTP, with the non-redundant protein sequence database from National Centre for Biotechnology Information (NCBI), as part of the generic ESTExplorer pipeline for systematic EST analysis and annotation.

Protein databases for 'parasitic nematodes' and 'non-nematodes' were generated in-house for similarity searches. The 'parasitic nematodes' group contains all available protein sequences for parasitic nematodes and ESTs from GenBank (17 February 2007), translated into peptide sequences, whereas the 'non-nematodes' database comprises amino acid sequences from the complete non-redundant protein database NR (17 February 2007) excluding those from nematodes. Homologues to rESTs were identified *via *comparisons against WormBase using BLASTX and the Parasite genome WU-BLAST2 Nematoda database (from the European Bioinformatics Institute) using BLASTN. Each EST of *D. viviparus *was assigned a 'statistically significant' gene homologue if the E-value from the BLAST output of the sequence alignment was <1e-05. The program SimiTri [22] was used for the comparison (at the amino acid sequence level) of *D. viviparus *rESTs with data in *C. elegans*, parasitic nematode and non-nematode protein sequence databases. SimiTri provides a two-dimensional display of relative similarity relationships among three different datasets.

From the peptides inferred from rESTs, secreted proteins were predicted using a combination of three programs, to minimize the number of false positive predictions. Firstly, SignalP 3.0 [35] was used to predict the presence of secretory signal peptides and signal anchors for each predicted rEST proteins. A signal sequence was considered present when it was predicted both by the artificial neural network and the hidden Markov model prediction approaches (SignalPNN and SignalP-HMM, available as options within SignalP). In order to exclude the erroneous prediction of putative transmembrane (TM) sequences as signal sequences, TMHMM [36], a membrane topology prediction program, was then applied. We further validated the list of secreted proteins, using extracellular localization, employing PSORT [37].

*Note: *The final set of quality ESTs reported in this paper 15 are available in the EMBL, GenBank and DDJB databases under accession numbers EV849926 – EV854361.

## Authors' contributions

SR, SHN and RBG conceived and designed the research plan and participated in all aspects of data collection and analysis. SHN and SR analysed, and SHN, SR, MH and RBG interpreted the data. CS and TS provided adult worms of *D. viviparus*. RBG and MH isolated and purified RNA for cDNA library construction and supervised and coordinated the sequencing. All authors contributed to the analyses and the writing of the manuscript. All authors read and approved the final manuscript.

## Supplementary Material

Additional file 1 Comparison of 2258 rESTs from *Dictyocaulus viviparus *with *Caenorhabditis elegans* data (Wormpep v 167). The table also provides corresponding RNAi phenotypes in *C. elegans *and information on homologues in parasitic nematodes. Click here for file

Additional file 2 Secreted proteins predicted from rESTs from *Dictyocaulus viviparus*. Click here for file

Additional file 3 *Dictyocaulus viviparus *homologues in nematodes in clade V, including *Ancylostoma caninum, Ancylostoma ceylanicum, Necator americanus, Nippostrongylus brasiliensis, Haemonchus contortus, Ostertagia ostertagi, Teladorsagia circumcincta *and *Pristionchus pacificus*. Click here for file

Additional file 4 Mapped metabolic pathways in adult *Dictyocaulus viviparus *based on Kyoto Encyclopedia of Genes and Genomes (KEGG). The pathway mapping was carried out using the program KOBAS. Click here for file

Additional file 5 Gene Ontology mappings (using GO slim terms) for *Dictyocaulus viviparus *clusters generated using BLAST2GO program. Note that individual GO categories can have multiple mappings. Click here for file
