# Supplementary material for: A transcriptomic analysis of the adult stage of the bovine lungworm, Dictyocaulus viviparus
Source: BMC Genomics. 2007 Sep 5;8:311. doi: 10.1186/1471-2164-8-311 (PMC2131760; doi:10.1186/1471-2164-8-311)
Supplement: Additional file 2 — Secreted proteins predicted from rESTs from Dictyocaulus viviparus. [file 1471-2164-8-311-S2.pdf]

Additional Table 2: Secreted proteins predicted from rESTs from *Dictyocaulus viviparus.*

Number	EST sequence ID	Residue	Start	SP	Description (top NR hit)	E-value	% Identity (aa)	RNAi phenotype in *C. elegans*
1	DvContig8	222	-	24	secreted protein 5 precursor [*Ancylostoma caninum*]	2E-12	60/201 (29%)	None
2	DvContig13	407	-	12	Sperm-Specific family, class Q family member (ssq-2) [*Caenorhabditis elegans*]	1E-22	156/234 (66%)	locomotion abnormal (unc)
3	DvContig16	416	-	18	Protein phosphatase 2A homologues [*Caenorhabditis elegans*]	8E-84	154/337 (45%)	None
4	DvContig30:	144	M	19	Hypothetical protein CBG06429 [*Caenorhabditis briggsae*]	2E-14	6/85 (42%)	None
5	DvContig38	190	-	24	calumenin-like protein [*Caenorhabditis elegans*]	2E-71	140/173 (80%)	locomotion abnormal (unc)molt defect (Mlt)larval lethal (Let)larval arrest (Lva)clear (Clr)
6	DvContig39	172	M	20	emp24/gp25L/p24 family of membrane trafficking proteins] [*Caenorhabditis briggsae*]	5E-52	96/140 (68%)	None
7	DvContig76	357	-	20	[Peptidylglycine alpha-amidating monooxygenase] [*Caenorhabditis elegans*]	2E-107	177/297 (59%)	None
8	DvContig91	289	-	17	inorganic PYroPhosphatase family member (pyp-1)	1E-99	171/246 (69%)	embryonic lethal (Let)maternal sterile (Ste)larval lethal (Let)larval arrest (Lva)slow growth (Gro)

9	DvContig116	283	M	16	IG-domain protein family member (zig-3) [*Caenorhabditis elegans*]	7E-41	94/248 (37%)	None
10	DvContig117	138	-	16	nippocystatin [*Nippostrongylus brasiliensis*]	4E-48	84/122 (68%)	maternal sterile (Ste)
11	DvContig118	435	-	21	Nuclear Hormone Receptor family member (nhr-142) [*Caenorhabditis elegans*]	2E-60	133/375 (35%)	None
12	DvContig160	116	M	19	None	-	-	-
13	DvContig166	404	-	41	heat shock protein 70 [*Dirofilaria immitis*]	7E-77	136/187 (72%)	larval arrest (Lva)maternal sterile (Ste)Unclassifiedembryonic lethal (Let)transgene expression increased
14	DvContig200	102	M	20	None	-	-	-
15	DvContig216	343	-	38	Hypothetical protein CBG12561 [*Caenorhabditis briggsae*] [Contains Serine/Threonine protein kinases, catalytic domain]	8E-141	233/309 (75%)	None
16	DvContig225	341	M	17	cathepsin B-like cysteine protease 2 [*Parelaphostrongylus tenuis*]	7E-106	182/313 (58%)	None
17	DvContig231	121	-	24	None	-	-	-

18	DvContig259	220	M	19	secreted protein 5 precursor [*Ancylostoma caninum*]	2E-12	60/201 (29%)	None
19	DvContig325	247	-	15	Ribosomal Protein, Large subunit family member (rpl-19) [*Caenorhabditis elegans*]	2E-58	161/198 (81%)	larval arrest (Lva)maternal sterile (Ste)embryonic lethal (Let)embryonic terminal arrest variable emb (Emb)pleiotropic defects severe early emb (Emb)larval lethal (Let)
20	DvContig346	50	M	20	None	-		-
21	DvContig348	209	M	18	secreted protein ASP-2 [*Necator americanus*]	6E-12	67/223 (30%)	None
22	DvContig356	291	-	41	C25A1.8 [*Caenorhabditis elegans*] clec-87 - (C-type LECtin)	3E-31	76/191 (39%)	None
23	DvContig376	397	-	49	cysteine proteinase 5 [*Necator americanus*]	1E-92	170/338 (50%)	embryonic lethal (Let)locomotion abnormal (unc)larval arrest (Lva)
24	DvContig396	151	-	22	beta tubulin type 1 [*Ancylostoma caninum*]	2E-68	129/151 (85%)	pronuclear migration abnormal early emb (Emb)embryonic lethal (Let)
25	DvContig402	156	M	27	None	-	-	-
26	DvContig416	235	-	21	Hypothetical protein T09B4.7 [*Caenorhabditis elegans*] Contains Protein kinase domain	7E-55	100/178 (56%)	None

27	DvContig432	239	M	16	secreted protein 5 precursor [*Ancylostoma caninum*]	2E-15	65/208 (31%)	-
28	DvContig434	227	M	21	secreted protein 5 precursor [*Ancylostoma caninum*]	5E-15	59/198 (29%)	-
29	D.viviparus_10_E06	215	-	18	Seven B Two homolog family member (sbt-1) [*Caenorhabditis elegans*]	9E-71	131/210 (62%)	aldicarb resistant (Ric)
30	D.viviparus_10_H07	330	-	21	Hypothetical protein CBG13180 [*Caenorhabditis briggsae*] Contains Protein Kinase Domain	3E-28	85/248 (34%)	None
31	D.viviparus_11_C01	101	-	28	None	-	-	-
32	D.viviparus_2_C05	92	-	19	None	-	-	-
33	D.viviparus_2_D08	375	-	25	Hypothetical protein CBG18333 [*Caenorhabditis briggsae*]	1E-20	73/173 (42%)	-
34	D.viviparus_2_G05	82	M	22	None	-	-	-
35	D.viviparus_2_H04	376	-	22	metalloprotease 1 precursor [*Ancylostoma ceylanicum*]	1E-29	61/117 (52%)	None

36	D.viviparus_3_D06	216	-	19	None	-	-	-
37	D.viviparus_4_D06	290	-	16	Hypothetical protein CBG24164 [*Caenorhabditis briggsae*]	2E-13	54/159 (33%)	-
38	D.viviparus_4_F01	43	M	18	None	-	-	-
39	D.viviparus_5_H09	163	M	23	None	-	-	-
40	D.viviparus_6_D12	86	M	23	None	-	-	-
41	D.viviparus_7_B12	321	-	25	Sperm-Specific family, class Q family member (ssq-4) [*Caenorhabditis elegans*]	2E-03	63/152 (41%)	locomotion abnormal (unc)
42	D.viviparus_8_A01	332	-	21	secreted acetylcholinesterase [*Dictyocaulus viviparus*]	0E+00	315/327 (96%)	None
43	D.viviparus_8_C07	101	-	20	None	-	-	-
44	D.viviparus_9_B01	173	-	23	None	-	-	-

45	D.viviparus_12_A11	151	-	39	Hypothetical protein T22H2.6a [*Caenorhabditis elegans*] Granulin domain smart00277	1E-11	46/119 (38%)	None
46	D.viviparus_13_F01	92	-	23	None	-	-	-
47	D.viviparus_14_F01	113	-	23	None	-	-	-
48	D.viviparus_14_H04	254	-	20	Hypothetical protein CBG13511 [*Caenorhabditis briggsae*]	0.012	28/81 (34%)	-
49	D.viviparus_16_E10	365	-	18	Hypothetical protein C44B12.5 [*Caenorhabditis elegans*]	4E-23	91/239 (38%)	maternal sterile (Ste)sterile progeny (Stp)embryonic lethal (Let)
50	D.viviparus_18_C09	354	-	24	Hypothetical protein CBG20492 [*Caenorhabditis briggsae*] Peptidylglycine alpha-amidating monooxygenase	2E-84	154/295 (52%)	None
51	D.viviparus_18_D12	186	M	21	CG1749-PA [*Drosophila melanogaster*] Molybdopterin biosynthesis-related protein	5E-50	105/183 (57%)	None
52	D.viviparus_19_B07	338	-	36	MoLTing defective family member (mlt-9) [*Caenorhabditis elegans*]	4E-85	174/295 (58%)	molt defect (Mlt)locomotion abnormal (unc)paralyzed (Prl)sick (Sck)reduced brood sizeslow growth (Gro)late larval lethal (Let)larval arrest (Lva)

53	D.viviparus_19_E10	313	-	33	parasite pepsinogen [*Haemonchus contortus*]	2E-76	148/308 (48%)	cell death abnormal (Ced)
54	D.viviparus_20_A08	315	-	21	Sperm-specific protein ZC168.6 [*Caenorhabditis elegans*]	1E-12	42/100 (42%)	None
55	D.viviparus_20_D05	158	M	21	Hypothetical protein CBG02732 [*Caenorhabditis briggsae*]phospholipase A2	2E-40	85/142 (59%)	None
56	D.viviparus_20_E11	197	-	46	Hypothetical protein CBG21920 [*Caenorhabditis briggsae*]	3E-19	51/106 (48%)	None
57	D.viviparus_22_H06	109	-	23	None	-	-	None
58	D.viviparus_22_H07	39	-	23	None	-	-	None
59	D.viviparus_24_F10	343	M	19	Hypothetical protein CBG17607 [*Caenorhabditis briggsae*]PDZ domain	3E-23	89/324 (27%)	None
60	D.viviparus_24_F12	129	-	23	Hypothetical protein F44A2.5b [*Caenorhabditis elegans*]MIF4G domain containing protein.	4E-45	89/145 (61%)	None
61	D.viviparus_25_D08	358	-	39	cathepsin L 1 [*Dictyocaulus viviparus*]	2E-153	277/342 (80%)	embryonic lethal (Let)slow growth (Gro)locomotion abnormal (unc)

62	D.viviparus_26_C06	34	-	17	None	-	-	-
63	D.viviparus_26_D04	240	-	35	Hypothetical protein F28D1.8 [*Caenorhabditis elegans*] Contains IG domain	6E-10	33/91 (36%)	-
64	D.viviparus_26_D09	51	M	15	None	-	-	-
65	D.viviparus_26_F06	158	M	21	phospholipase A2 [*Caenorhabditis briggsae*]	2E-40	85/142 (59%)	None
66	D.viviparus_28_B12	67	M	41	None	-	-	-
67	D.viviparus_30_D10	137	-	18	Hypothetical protein CBG18309 [*Caenorhabditis briggsae*]	4E-13	36/87 (41%)	-
68	D.viviparus_30_H04	206	M	13	GRound-Like (grd related) family member (grl-5) [*Caenorhabditis elegans*]	2E-27	64/98 (65%)	None
69	D.viviparus_31_F11	118	-	18	None	-	-	-
70	D.viviparus_41_C07	233	-	58	ARp2/3 compleX component family member (arx-7) [*Caenorhabditis elegans*]	5E-40	80/121 (66%)	embryonic lethal (Let)locomotion abnormal (unc)sick (Sck)larval arrest (Lva)

71	D.viviparus_42_C05	92	M	18	None	-	-	-
72	D.viviparus_33_A03	284	M	24	Hypothetical protein CBG11012 [*Caenorhabditis briggsae*] contains Leucine rich repeats	5E-41	99/222 (44%)	None
73	D.viviparus_35_B12	177	M	16	None	-	-	-
74	D.viviparus_46_H03	219	M	20	None	-	-	None
75	D.viviparus_43_F07	186	M	17	Phospholipase A2-like protein Y52B11A.8 [*Caenorhabditis elegans*]	2E-22	43/100 (43%)	None
76	D.viviparus_47_F01	175	-	23	None	-	-	-
77	D.viviparus_48_A09	208	-	22	heat shock protein 20 [*Haemonchus contortus*]	5E-30	60/124 (48%)	Unclassifiedlife span abnormal (Age)
78	D.viviparus_50_H11	335	-	22	Defective spermatogenesis protein 8 [*Caenorhabditis elegans*]	3E-21	73/227 (32%)	None
79	D.viviparus_51_F01	206	M	13	GRound-Like (grd related) family member (grl-5) [*Caenorhabditis elegans*]	2E-27	64/98 (65%)	None

80	D.viviparus_52_B03	338	-	27	Hypothetical protein ZK1128.3 [*Caenorhabditis elegans*]	2E-12	58/194 (29%)	-
81	D.viviparus_52_F07	133	-	24	Mechanosensory abnormality protein 12 [*Caenorhabditis elegans*] alpha-tubulin MEC-12 [*Caenorhabditis elegans*]	8E-51	95/103 (92%)	Only Secondary targets
82	D.viviparus_52_F12	160	-	21	major sperm protein [*Dictyocaulus viviparus*]	2E-66	122/126 (96%)	fat content increased
83	D.viviparus_52_G05	230	-	22	Ribosomal Protein, Large subunit family member (rpl-19) [*Caenorhabditis elegans*]	6E-55	161/198 (81%)	larval arrest (Lva)maternal sterile (Ste)embryonic lethal (Let)embryonic terminal arrest variable emb (Emb)pleiotropic defects severe early emb (Emb)larval lethal (Let)
84	D.viviparus_53_A07	301	M	21	Yeast glc seven-like phosphatases protein 3 [*Caenorhabditis elegans*] putative serine/threonine phosphatase [*O.dentatum*]	3E-100	162/284 (57%)	None
85	D.viviparus_56_G03	274	-	15	acyl-CoA dehydrogenase [*Caenorhabditis briggsae*]	9E-110	195/261 (74%)	None
